# Influences of Daily Life Habits on Risk Factors of Stroke Based on Decision Tree and Correlation Matrix

**DOI:** 10.1155/2020/3217356

**Published:** 2020-06-01

**Authors:** Zeguo Shao, Yuhong Xiang, Yingchao Zhu, Aiqin Fan, Peng Zhang

**Affiliations:** ^1^School of Medical Instrumentation, Shanghai University of Medicine &Health Sciences, Shanghai 201318, China; ^2^Center for Intelligent Medical Electronics (CIME), Fudan University, Shanghai 201318, China; ^3^Nursing Department, Shanghai Pudong New District Zhoupu Hospital, Shanghai 201318, China; ^4^Pudong New Area Lingqiao Community Health Service Center, Shanghai 200137, China; ^5^School of Clinical Medicine, Shanghai University of Medicine & Health Sciences, Shanghai 201318, China; ^6^Shanghai General Practice Medical Education and Research Center, Shanghai 201318, China

## Abstract

**Purpose:**

To explore the influences of smoking, alcohol consumption, drinking tea, diet, sleep, and exercise on the risk of stroke and relationships among the factors, present corresponding knowledge-based rules, and provide a scientific basis for assessment and intervention of risk factors of stroke.

**Methods:**

The decision tree C4.5 algorithm was optimized and utilized to establish a model for stroke risk assessment; then, the main risk factors of stroke (including hypertension, dyslipidemia, diabetes, atrial fibrillation, body mass index (BMI), history of stroke, family history of stroke, and transient ischemic attack (TIA)) and daily habits (e.g., smoking, alcohol consumption, drinking tea, diet, sleep, and exercise) were analyzed; corresponding knowledge-based rules were finally presented. Establish a correlation matrix of stroke risk factors and analyze the relationship between stroke risk factors.

**Results:**

The accuracy of the established model for stroke risk assessment was 87.53%, and the kappa coefficient was 0.8344, which was superior to that of the random forest and Logistic algorithm. Additionally, 37 knowledge-based rules that can be used for prevention of risk factors of stroke were derived and verified. According to in-depth analysis of risk factors of stroke, the values of smoking, exercise, sleep, drinking tea, alcohol consumption, and diet were 6.00, 7.00, 8.67, 9.33, 10.00, 10.60, and 10.75, respectively, indicating that their influence on risk factors of stroke was reduced in turn; on the one hand, smoking and exercise were strongly associated with other risk factors of stroke; on the other hand, sleep, drinking tea, alcohol consumption, and diet were not firmly associated with other risk factors of stroke, and they were relatively tightly associated with smoking and exercise.

**Conclusions:**

Establishment of a model for stroke risk assessment, analysis of factors influencing risk factors of stroke, analysis of relationships among those factors, and derivation of knowledge-based rules are helpful for prevention and treatment of stroke.

## 1. Introduction

Stroke is an acute cerebrovascular disease, associating with the characteristics of high morbidity, high disability, and high mortality. It is a refractory disease that imposes a major threat to human health and life [1]. At present, there are no effective treatments for stroke. Prevention is still the most feasible strategy to reduce the harm of stroke and reduce its social burden, especially with respect to high global incidence and potential risk factors of stroke [2]. The risk factors of stroke are divided into intervention factors (e.g., smoking, alcohol consumption, and body mass index (BMI)) and nonintervention factors (e.g., age, gender, ethnicity, and genetic attributes) according to whether the risk can be changed through intervention [3]. Hence, studying the intervention factors is of great significance for the prevention of stroke. In addition, we previously found that the interventional risk factors for stroke appeared more in people's daily lives and behavioral habits [4, 5]. Unhealthy lifestyles can trigger or increase the risk of stroke, and moderate lifestyle changes may reduce the risk of stroke as well [6]. Therefore, numerous scholars suggested that further studies should be carried out to provide effective interventions to guide and improve people's lifestyle, so as to reduce the risk and incidence of stroke [7–9]. However, in 2019, Altobelli et al. analyzed the relevant literature and found that research in this area was conducted in only a limited number of developed countries, and there were very few reports on the impact of lifestyle and dietary habits on risk factors of stroke [10]. In China, Huang et al. conducted relevant research and demonstrated that a healthy lifestyle (high fruit intake, quitting smoking, doing housework, and good sleep quality) may reduce the chance of recurrence of first-onset ischemic stroke [11]. Although the risk factors of stroke in daily life habits are not the main risk factors of stroke, they are closely associated with the main risk factors [12].

The present study was aimed at the Chinese population, and large-scale and multidimensional stroke data were collected through modern information technology. The optimized decision tree algorithm was used to analyze risk factors of stroke in daily life habits, derive knowledge-based rules, and establish a model for stroke risk assessment to analyze relationships among risk factors of stroke.

## 2. Materials and Methods

### 2.1. Data Collection and Pretreatment

**Figure 1:**
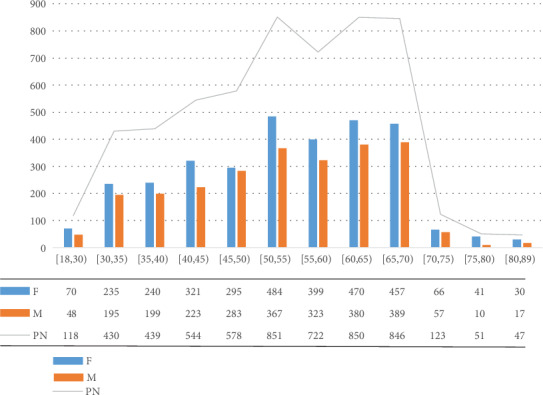
Distribution of age- and gender-based data.

We established a whole-course stroke management network system via collection of large-scale data from Shanghai suburban population, involving nearly 10,000 people, in which 5599 valid data were finally acquired. The data included subjects' demographic characteristics, physical examination, family medical history, treatment history, personal diet and lifestyle habits, sleep and breathing, psychological status, quality of life, and stroke knowledge. In order to facilitate classification of stroke, we also designed a rapid stroke screening form and performed statistical analysis. We preliminarily extracted and integrated data and determined 16 risk factors of stroke for further analysis. As shown in Table 1, among 5599 data collected, there were 2491 males and 3108 females, subjects' minimum and maximum age were 18 and 89 years old, respectively. The age- and gender-based data are shown in Figure 1.

As illustrated in Figure 1, [18,30) indicates that age is 18 years old or older and less than 30 years old; F and M denote female and male, respectively; and PN is the number of individuals.

**Figure 2:**
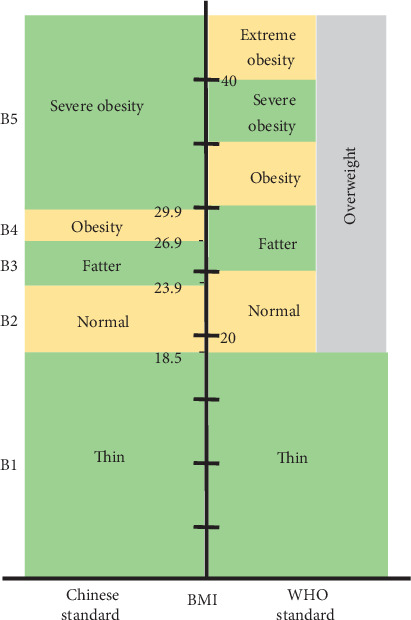
BMI classification.

The present research analyzed the risk factors of smoking, alcohol consumption, drinking tea, diet, sleep, sport, and BMI. The above-mentioned factors were defined as follows:

- Smoking: those who have smoked for 6 months or more in their lifetime were marked as “y”; otherwise, they are denoted as “n”
- Alcohol consumption: those who have drunk no less than twice/week and no less than 80 ml each time were marked as “y”; otherwise, they were denoted as “n”
- Drinking tea: those who have drunk tea at least 3 days/week were marked as “y”; otherwise, they were denoted as “n”
- Diet: the daily food ingredients are mainly sugars, fats, or proteins, which were marked with “C1,” “C2,” and “C3,” respectively
- Sport: those who have exercised sport more than 3 times/week and more than 30 min each time, demonstrating regular level of sport, marked as “C1”; those who have exercised sport 2-3 times/week, and 10-30 min each time, reflecting medium level of sport, marked as “C2”; those who have exercised less than or equal to 1 time/week and less than 10 min each time, indicating lower level of sport, marked “C3”
- BMI: since the WHO standards are not highly appropriate for Chinese people, the Chinese Reference Standards were formulated with reference to the WHO standards and are divided into five types: B1, B2, B3, B4, and B5 (Table 2)
- Sleep: duration of sleep in different ages can be divided into three types: very short-term, medium-term, and very long-term, which could be labelled as TS, TB, and TL, respectively, as shown in Figure 2

According to the rapid screening of risk factors of stroke (including hypertension, dyslipidemia, diabetes, atrial fibrillation, smoking history, BMI, sport, stroke history, family history of stroke, and transient ischemic attack (TIA)), refer to the Guidelines for Screening, Prevention and Control of Ischemic Stroke presented by the Ministry of Health of China (hereinafter referred to the guidelines), this study classified stroke risk into H, M, L, N, T, and Y levels, as summarized in Table 3.

### 2.2. Decision Trees

The decision tree is a popular, logic-based, easily interpretable, straightforward, and widely applicable method [13]. The classic decision tree algorithms include ID3, C4.5, and CART. In contrast to ID3, which can only handle discrete variables, C4.5 and CART can handle continuous variables, and they are not sensitive to incomplete data. In addition, the CART generates binary trees and the C4.5 algorithm generates multiple branches. Decision trees can generate interpretable knowledge rules, which can express relationship between factors. This is in line with our goal to explore relationships among the risk factors of stroke. Therefore, the C4.5 algorithm was selected in the current research. Details of the C4.5 algorithm were described in the following.

#### 2.2.1. C4.5 Algorithm

In 1992, Ross Quinlan developed the C4.5 decision tree algorithm [14]. C4.5 constructs a decision tree as a learning model from the data samples. The divide-and-conquer approach is adopted for construction of decision tree models using a measure called information gain to select the attribute from the dataset for the tree.

*(1) Information Gain*. Suppose that there are *C* categories of data in the sample dataset *D*. The information entropy formula is as follows:

(1) $$\begin{array}{c} \text{Info }\left(D\right)=-\overset{c}{\underset{i=1}{\sum }}p_{i}\times \log_{2}\left(p_{i}\right), \end{array}$$

where *D* represents the training dataset, *C* denotes the data class number, and *p*_*i*_ represents the ratio of the sample number in class *i* to all samples. When the attribute *A* is chosen as the node of the decision tree, the information entropy after the action of feature *A* is as follows:

(2) $$\begin{array}{c} {\text{Info}}_{A}\left(D\right)=-\overset{k}{\underset{j=1}{\sum }}\frac{\left|D_{j}\right|}{\left|D\right|}\times \text{Info}\left(D_{j}\right), \end{array}$$

where *k* represents the data samples *D* divided into *k* parts.

*(2) Gain Ratio*. The information gain represents the value of the information entropy that the dataset *D* decreases after the action of the feature *A*. The formula is as follows:

(3) $$\begin{array}{c} \text{Gain }\left(A\right)=\text{Info }\left(D\right)-{\text{Info}}_{A}\left(D\right). \end{array}$$

The information gain ratio is given by

(4) $$\begin{array}{c} \text{Gain Ratio}\left(A\right)=\frac{\text{Gain}\left(A\right)}{{\text{Info}}_{A}\left(D\right)}. \end{array}$$

#### 2.2.2. Improvement and Implementation of C4.5 Algorithm

We used a decision tree algorithm to analyze the above-mentioned 16 risk factors of stroke (see Table 1). The decision tree is generated using the J48 (C4.5 algorithm implementation) in the Weka classifier algorithm. The confidence factor for the pruning is set to 0.25, and the minimum number of instances per leaf (minNumObj) is set to 1. The 10-fold cross-validation is additionally used to select and evaluate the model.

**Figure 3:**
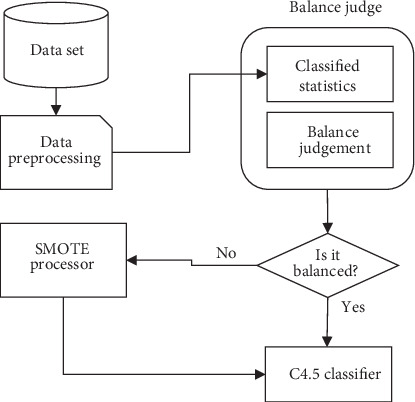
SMOTE+C4.5 classification model.

In order to solve imbalanced data problem and improve the robustness of the system, we, in the current study, presented SMOTE algorithm to improve the model. The SMOTE algorithm is an intelligent oversampling technique for unbalanced datasets proposed by Chawla et al. in 2002. It can effectively improve the overfitting phenomenon caused by traditional oversampling techniques and solve the problem of biased classification results. As illustrated in Figure 3, after classified dataset is preprocessed for equilibrium judgment, the number of records in each class is first counted to find out the maximum value (max) and minimum value (min) of the number of records and then quotient max and min, if max/min < 3. After the dataset is judged to be balanced, it is directly entered into the C4.5 classifier for classification. Otherwise, it is judged that the dataset is unbalanced and is entered into the SMOTE processor: first, the entire dataset is sampled, the sampling method is nonrepeatable sampling, the number is equal to the number of datasets, each record is randomly sorted, and then, SMOTE is used to generate new minority data. The effects of operations, such as filtering and sorting preprocessing on the SMOTE algorithm, are eliminated to ensure that the data obtained by SMOTE is obtained by randomly combining the major data and the minor data to avoid overfitting caused by the data generated by SMOTE only from the minor data. Then, the data are entered into the classification module.

## 3. Results

**Figure 4:**
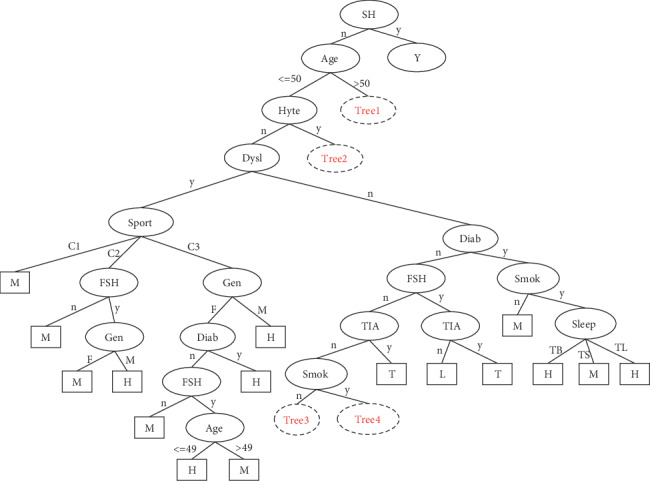
A decision tree to classify risk factors of stroke.

**Figure 5:**
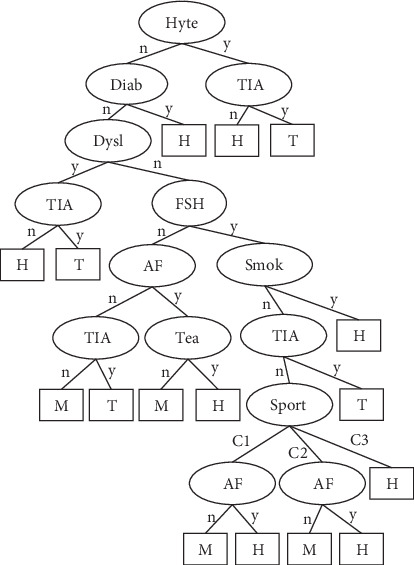
Decision tree #1 to classify risk factors of stroke.

**Figure 6:**
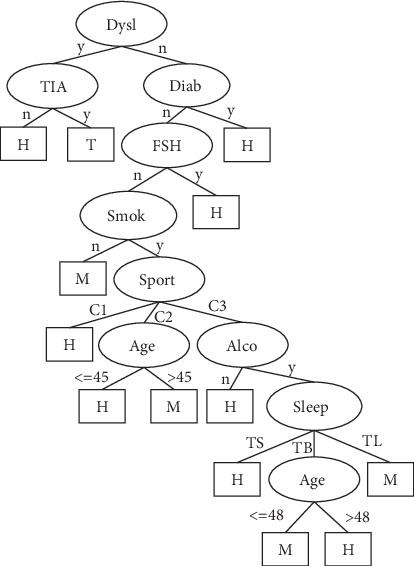
Decision tree #2 to classify risk factors of stroke.

**Figure 7:**
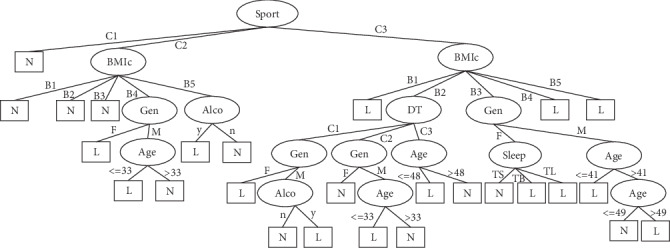
Decision tree #3 to classify risk factors of stroke.

**Figure 8:**
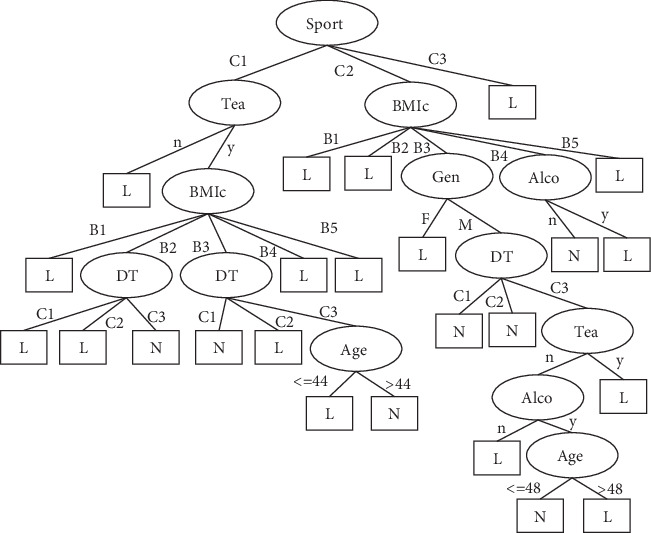
Decision tree #4 to classify risk factors of stroke.

The number of leaves of the tree was 98, while the size of the tree was 171 (Figures 4–8). The performance indexes of the tree are as follows: classification accuracy: 87.5281%; kappa statistic: 0.8344; mean absolute error: 0.0567; and root-mean-square error: 0.175.

To assess the performance of the proposed system for stroke risk classification, precision, recall, accuracy, and kappa were calculated, and 10-fold cross-validation was used. Equations (5)–(8) were presented to calculate precision, recall, accuracy, and kappa, respectively.

(5) $$\begin{array}{c} \text{Precision}=\frac{\text{TP}}{\text{TP}+\text{FP}}, \end{array}$$

(6) $$\begin{array}{c} \text{Recall}=\frac{\text{TP}}{\text{TP}+\text{FN}}, \end{array}$$

(7) $$\begin{array}{c} \text{Accuracy}=\frac{\text{TP}+\text{TN}}{\text{TP}+\text{TN}+\text{FP}+\text{FN}}, \end{array}$$

(8) $$\begin{array}{c} \text{Kappa}=\frac{p_{o}-p_{e}}{1-p_{e}}. \end{array}$$

Precision represents the correct positive prediction ratio to the whole positive samples. Recall is the correct positive prediction ratio to the whole positive predictions. Accuracy is correct prediction ratio to the whole predictions. True positives (TPs) are positive cases that are correctly predicted as positive. False negatives (FNs) are positive cases that are incorrectly predicted as negative. True negatives (TNs) are negative cases that are correctly predicted as negative. False positives (FPs) are negative cases that are incorrectly predicted as positive. Meanwhile, kappa offers a more robust estimated performance of the proposed system compared with a simple agreement and gives an overall evaluation of all the cases. *p*_*o*_ is the relative observed agreement among the proposed system and the physician analysis, and *p*_*e*_ is the hypothetical probability of chance agreement.

Table 4 presents the confusion matrix of the classification result using optimized C4.5 algorithm. In order to evaluate the performance of the optimized C4.5 algorithm, the random forest and Logistic algorithm were implemented for making comparison. Random forests or random decision forests are an ensemble learning method for classification, regression, and other tasks that operate by constructing a multitude of decision trees at training time and outputting the class that is the mode of the classes (classification) or mean prediction (regression) of the individual trees [15]. Logistic regression is a generalized linear regression analysis model, commonly used in data mining, automatic disease diagnosis, economic prediction, and other fields. The Logistic regression is good at analyzing linear relationships, and analyzing nonlinear relationships is worse than decision trees. In addition, it is sensitive to extreme values and easily affected by extreme values, and the decision tree performs better in this respect [16].

In the current study, the number of trees in the random forest was set to 100, and for each tree, the minimum number of instances for each leaf was set to 1. The Ridge value in the Logistic was set to 1.0*E* − 8, and the maximum number of iterations to perform was set to -1. They all use tenfold cross-validation like decision trees. Tables 5 and 6 summarize the confusion matrix of classification results using random forest and Logistic algorithm, respectively.

**Figure 9:**
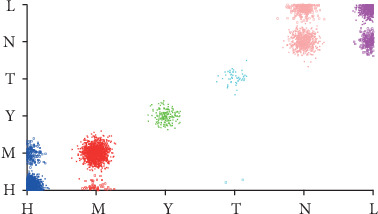
Illustration of errors of the optimized C4.5 algorithm.

**Figure 10:**
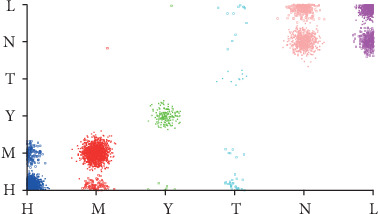
Illustration of errors of the random forest algorithm.

**Figure 11:**
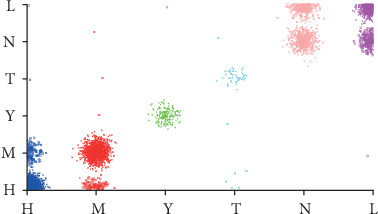
Illustration of errors of the Logistic algorithm.

Regardless of accuracy or kappa value, the optimized C4.5 is the highest among the three algorithms. The recall of the risk type “T” could achieve only 0.208 using the random forest algorithm, which was noticeably lower than 0.962 using the C4.5 algorithm. Figures 9–11 demonstrate that misclassification rate of risk type “T” is the lowest in optimized C4.5 algorithm among the three algorithms.

Corresponding knowledge-based rules can be deduced from the decision tree. There were 98 knowledge-based rules deduced from the present case. There are 37 rules related to the 6 daily living habits (smoking, alcohol consumption, drinking tea, diet, sleep, and sport), which are illustrated in the Supplementary Information (available here).

## 4. Discussion

According to the previous decision tree, the average depth and frequency of each risk factor in the decision tree were calculated, as shown in Table 7. Values of risk factors for stroke (stroke history, hypertension, dyslipidemia, diabetes, family history of stroke, TIA, smoking, atrial fibrillation, exercise, sleep, gender, BMI, drinking tea, age, and alcohol consumption) were increased, indicating that their influence on risk factors of stroke was relatively reduced. Simultaneously, the impact of daily living habits on risk factors of stroke was relatively insignificant, demonstrating that the influence of lifestyle habits and diet on risk factors of stroke is indirect.

We further analyzed the above-mentioned 98 knowledge-based rules for risk factors of stroke, in which risk factors were extracted from the knowledge-based rules. Within each set, the sum of the reciprocals of factors was used to represent the weight of each factor. All factor sets and their weights will be described in the Supplementary Information. Within each set, every two factors formed a factor pair; the same factor pairs were weighted and summed together to form a factor-based relationship matrix, as shown in Table 8.

As illustrated in Table 8, it was unveiled that the risk factors of stroke, such as stroke history (SH), hypertension (Hyte), dyslipidemia (Dysl), diabetes (Diab), and age (Age), have the highest correlation. Of the 6 daily habit factors we examined (smoking, alcohol consumption, tea, diet, sleep, and exercise), only the correlation of smoking (Smok) and sport (Sport) was higher than the average (1.95). This indicates that alcohol consumption, drinking tea, diet, and sleep are not strongly correlated with other factors. In addition, regarding this weak correlation, the correlation values of alcohol consumption, drinking tea, diet, sleep, smoking, and sport were close to those of strong correlation categories (SH, Hyte, Dysl, Diab, and Age), as shown in Table 9.

### 4.1. Smoking and Sport

**Figure 12:**
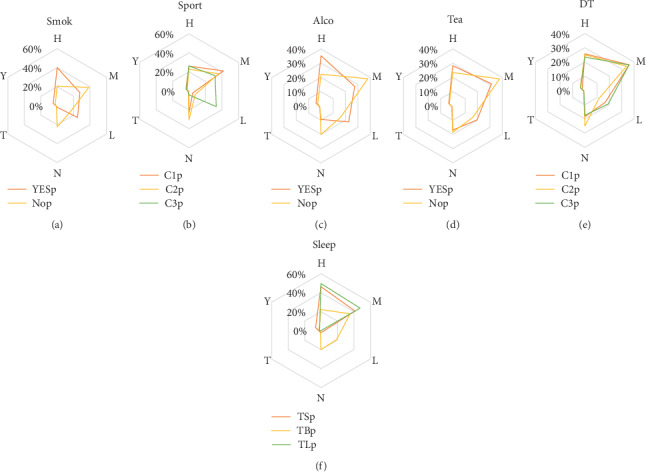
Radar charts illustrating the effects of daily life habits on risk factors of stroke.

Of the 37 knowledge-based rules mentioned above, 30 rules included a “smoking” factor, suggesting that smoking significantly increases the risk factors of stroke. Yamagishi et al. demonstrated that smoking increases the risk of stroke in patients with hypertension [17], which is in line with our findings. In addition, the radar chart of the risk ratio of smoking to nonsmoking is also illustrated by Figure 12(a).

Of the 37 knowledge-based rules mentioned above, 35 contained “sport.” As displayed in Figure 12(b), there is no significant difference in the impact of high-intensity and medium-intensity exercise on risk factors of stroke. Exercise is the most common factor affecting the risk of stroke, and moderate exercise helps prevent stroke, which is consistent with the results of McDonnell et al.'s study [18].

Additionally, 28 knowledge-based rules contained both “smoking” and “sport” factors, indicating that smoking and sport are closely associated together, and further, doing exercise by smokers is beneficial to reduce the risk of stroke.

### 4.2. Alcohol Consumption and Drinking Tea

It was noted that individuals who drink alcohol have a significantly higher risk of stroke than nonalcohol consumers (Figure 12(c)). This is in line with Hu et al.'s outcome that heavy drinking can increase the risk of stroke, while moderate drinking has insignificant influence on the risk of stroke [19]. However, it is not an independent factor and is typically associated with hypertension, diabetes, and hypercholesterolemia.

Knowledge-based rules showed that drinking tea has no direct effect on the risk of stroke (Figure 12(d)), and similar to alcohol consumption, it can be related to BMI. Sosa et al. demonstrated that tea is highly beneficial to reduce the risk of stroke in obese people [20]. Zhang et al. conducted experiments on mice and concluded that drinking tea has a neuroprotective effect on hemorrhagic stroke [21]. In addition, we found that “tea = y” and “alco = y” do not simultaneously appear in the same rule in the present study, and the correlation value of 0.14 (Table 8) between them is also very insignificant, indicating that drinking tea and alcohol consumption have simultaneously no effect on the risk of stroke.

### 4.3. Diet

As shown in Figure 12(e), the effects of the three types of diet (mainly sugar, fat, and protein) on risk of stroke are not significantly different. According to the rules, these types are more concentrated in the “H” and “M” types, demonstrating that dietary structure has a certain influence on individuals with high risk of stroke. In addition, from the perspective of correlation value (Table 8), it has a relatively higher correlation with other factors compared with alcohol consumption, drinking tea, and sleep.

### 4.4. Sleep

As displayed in Figure 12(f), the risk of stroke is lower when duration of sleep is appropriate. Very long or short duration of sleep is not conducive to avoid the risk of stroke, which is consistent with Huang et al.'s findings, expressing that a good sleep quality helps reduce the risk of stroke [11, 22]. From the perspective of rules, sleep is associated with smoking, alcohol consumption, and sport, and from the perspective of correlation, sleep, smoking, and exercise are relatively correlated together. People who exercise less and are obese have an increased risk of stroke, if the duration of their sleep is extremely long. People who exercise less, as well as being smokers, and alcohol drinkers have a higher risk of stroke, if the duration of their sleep would be lower than normal level.

As shown in Figure 12(a), “YESp” stands for “smoking” and “Nop” stands for “nonsmoking.” As illustrated in Figure 12(b), “C1p,” “C2p,” and “C3P” represent three kinds of exercise: “C1,” “C2,” and “C3.” In Figure 12(c), “YESp” stands for “drinking,” and “Nop” denotes “no drinking.” As displayed in Figure 12(d), “YESp” stands for “drinking tea,” and “Nop” represents “no tea drinking.” As depicted in Figure 12(e), “C1p,” “C2p,” and “C3p” represent “C1,” “C2,” and “C3,” respectively. As illuminated in Figure 12(f), “TSp,” “TBp,” and “TLp” denote “TS,” “TB,” and “TL,” respectively.

## 5. Conclusions

In the present study, we optimized the decision tree C4.5 algorithm to assess and analyze risk factors of stroke (stroke history, hypertension, dyslipidemia, diabetes, family history of stroke, TIA, smoking, atrial fibrillation, sport, sleep, gender, BMI, drinking tea, age, alcohol consumption, and diet) via 5599 valid data collected. The classification result showed to have an accuracy of 87.5281% and a kappa coefficient of 0.8344. It also was noted that classification performance was higher than that of the random forest and Logistic algorithm. Then, we focused on 6 factors influencing daily life, such as smoking, alcohol consumption, drinking tea, sleep, and sport, and presented a series of knowledge-based rules that are conducive to guide patients to adjust individuals' living habits. With further analysis of decision tree and knowledge-based rules, the independent influence of each factor and the relationship between the factors were analyzed. Different from other studies, we analyzed the relationship between smoking and exercise, among alcohol consumption, drinking tea, and BMI, among diet, sport, and BMI, and among sleep, sport, smoking, and alcohol consumption and found that although these daily living habits cannot directly determine the risk of stroke (with low independent influence) they could be used to intervene the risk factors of stroke. On the one hand, smoking and exercise were strongly associated with other risk factors of stroke; on the other hand, sleep, drinking tea, alcohol consumption, and diet were not firmly associated with other risk factors of stroke, and they were relatively tightly associated with smoking and exercise. However, further research needs to be conducted to indicate whether smoking and exercise play a significant role in the risk of stroke in daily habits.

## Figures and Tables

**Table 1:** Subjects' clinical data.

Type of data	Risk factor of stroke	Field	Data distribution
Clinical diagnosis	Hypertension	Hyte	y: 1242, n: 3782, uncertain: 575
	Dyslipidemia	Dysl	y: 511, n: 4508, uncertain: 580
	Diabetes	Diab	y: 403, n: 4618, uncertain: 578
	Atrial fibrillation	AF	y: 75, n: 4940, uncertain: 584

Medical history and family history	Family history of stroke	FSH	y: 449, n: 4460, uncertain: 690
	History of stroke	SH	y: 165, n: 4730, uncertain: 704
	TIA	TIA	y: 95, n: 4350, uncertain: 1154

Demographic information	Gender	Gen	M: 2491, F: 3108
	Age	Age	Refer to Figure 1

Physical examination	BMI	BMIc	B1: 205, B2: 2926, B3: 1760, B4: 520, B5: 150, uncertain: 38

Daily habits	Smoking	Smok	y: 1192, n: 4379, null: 28
	Alcohol consumption	Alco	y: 1065, n: 4500, null: 34
	Drinking tea	Tea	y: 1563, n: 3997, null: 39
	Diet	DT	C1: 2812, C2: 263, C3: 2181, null: 370
	Sleep	Sleep	TS: 366, TB: 4958, BL: 205, null: 70
	Exercise sport	Sport	C1: 1518, C2: 1624, C3: 2275, null: 182

“y” means “yes,” “n” indicates “no,” and definitions of the types of BMI, diet, sleep, and exercise are presented in Figure 1. In Figure 1, we sometimes use fields to represent their corresponding stroke risk factors.

**Table 2:** Sleep classification.

Age	Duration of sleep (hours)	Mark
<3 (months)	<14	TS
	14~17	TB
	>17	TL

1~2 (years old)	<11	TS
	11~14	TB
	>14	TL

6~13 (years old)	<9	TS
	9~11	TB
	>11	TL

14~17 (years old)	<8	TS
	8~10	TB
	<10	TL

18~64 (years old)	<6	TS
	6~10	TB
	<10	TL

>64 (years old)	<7	TS
	7~8	TB
	<8	TL

**Table 3:** Definition of different levels of risk factors of stroke.

Type	Definition
Y	Have a history of stroke.
T	Has a previous transient ischemic attack.
H	The major risk factors defined in the guidelines are 2 items or more, or the major risk factors include 1 item, and the secondary risk factors involve 2 items or more.
M	The major risk factors defined in the guidelines include 1 item, and the secondary risk factors involve less than 2 items.
L	The main risk factors defined in the guidelines include 0 item, and the secondary risk factors involve 2 items or more.
N	The main risk factors defined in the guidelines include 0 item, and the secondary risk factors involve less than 2 items.

**Table 4:** Confusion matrix achieved by the optimized C4.5 algorithm.

	Risk level analyzed by optimized C4.5 algorithm						Recall
	H	M	Y	T	N	L	
Risk level analyzed by physicians							
H	**1288**	127	0	0	0	0	0.910
M	44	**1502**	0	0	0	0	0.972
Y	0	0	**165**	0	0	0	1.000
T	2	0	0	**51**	0	0	**0.962**
N	0	0	0	0	**679**	255	0.727
L	0	0	0	0	182	**596**	0.766
Precision	0.966	0.922	1.000	1.000	0.789	0.700	
Accuracy	87.53%						
Kappa	0.8344						

**Table 5:** Confusion matrix achieved by the random forest algorithm.

	Risk level analyzed by random forest algorithm						Recall
	H	M	Y	T	N	L	
Risk level analyzed by physicians							
H	**1300**	115	0	0	0	0	0.919
M	72	**1473**	0	0	1	0	0.953
Y	6	0	**158**	0	0	1	0.958
T	24	6	0	**11**	3	9	**0.208**
N	0	0	0	0	**699**	235	0.748
L	0	0	0	0	239	**539**	0.693
Precision	0.927	0.924	1.000	1.000	0.742	0.688	
Accuracy	85.46%						
Kappa	0.8063						

**Table 6:** Confusion matrix achieved by the Logistic algorithm.

	Risk level analyzed by Logistic						Recall
	H	M	Y	T	N	L	
Risk level analyzed by physicians							
H	**1289**	124	0	1	0	1	0.911
M	97	**1446**	1	1	1	0	0.935
Y	0	0	**164**	0	0	1	0.994
T	5	0	1	**46**	1	0	0.868
N	0	0	0	0	**690**	244	0.739
L	0	1	0	0	214	**563**	0.724
Precision	0.927	0.920	0.988	0.958	0.762	0.696	
Accuracy	85.83%						
Kappa	0.8119						

**Table 7:** Values of risk factors for stroke.

Risk factors	Depth/frequency															Average depth
	0	1	2	3	4	5	6	7	8	9	10	11	12	13	14	
SH	1															0.00
Hyte			2													2.00
Dysl				2	1											3.33
Diab				4	2		1									3.71
FSH						4		1								5.40
TIA				1	1	1	2	2								5.43
Smok						1	2	1								6.00
AF							7			2						6.67
Sport					1			1	3							7.00
Sleep							1			1		1				8.67
Gen						1	1				3	2				9.00
BMI										3	1					9.25
Tea								1		1			1			9.33
Age		1							2		1	3	3		1	10.00
Alco									1		2		1	1		10.60
DT											1	3				10.75

**Table 8:** A factor-based relationship matrix.

	SH	Hyte	Dysl	Diab	FSH	TIA	Smok	AF	Sport	Sleep	Gen	BMIc	Tea	Age	Alco
Hyte	6.84														
Dysl	6.34	6.34													
Diab	5.71	5.71	5.46												
FSH	5.71	4.95	4.95	4.64											
TIA	3.91	3.91	3.66	3.46	3.29										
Smok	4.16	4.16	4.16	4.16	3.85	3.03									
AF	0.45	0.45	0.45	0.45	0.45	0.33	0.20								
Sport	4.49	4.49	4.49	3.82	3.98	2.90	3.44	0.20							
Sleep	0.42	0.42	0.42	0.42	0.27	0.08	0.42	0.00	0.27						
Gen	1.74	1.74	1.74	1.43	1.43	1.05	1.05	0.00	1.74	0.08					
BMIc	2.17	2.17	2.17	2.17	2.17	2.17	2.17	0.00	2.17	0.08	1.05				
Tea	0.60	0.60	0.60	0.60	0.60	0.48	0.48	0.13	0.48	0.00	0.22	0.38			
Age	6.84	6.84	6.34	5.71	4.95	3.91	4.16	0.45	4.49	0.42	1.74	2.17	0.60		
Alco	0.70	0.70	0.70	0.70	0.70	0.40	0.70	0.00	0.70	0.19	0.22	0.40	0.14	0.70	
DT	0.96	0.96	0.96	0.96	0.96	0.96	0.96	0.00	0.96	0.00	0.62	0.86	0.38	0.96	0.22

**Table 9:** Factors with higher correlation values than the mean values within the group.

Smok		Sport		Sleep		Tea		Alco		DT	
Factors	Correlation	Factors	Correlation	Factors	Correlation	Factors	Correlation	Factors	Correlation	Factors	Correlation
SH	4.16	SH	4.49	SH	0.42	SH	0.60	SH	0.70	SH	0.96
Hyte	4.16	Hyte	4.49	Hyte	0.42	Hyte	0.60	Hyte	0.70	Hyte	0.96
Dysl	4.16	Dysl	4.49	Dysl	0.42	Dysl	0.60	Dysl	0.70	Dysl	0.96
Diab	4.16	Age	4.49	Age	0.42	Age	0.60	Age	0.70	Age	0.96
Age	4.16	FSH	3.98	Diab	0.42	Diab	0.60	Diab	0.70	Diab	0.96
FSH	3.85	Diab	3.82	Smok	0.42	FSH	0.60	FSH	0.70	FSH	0.96
Sport	3.44	Smok	3.44	FSH	0.27	Smok	0.48	Smok	0.70	Smok	0.96
TIA	3.03	TIA	2.90	Sport	0.27	Sport	0.48	Sport	0.70	Sport	0.96
						TIA	0.48			TIA	0.96
										BMI	0.86

The effects of the 6 daily habits (smoking, alcohol consumption, drinking tea, diet, sleep, and exercise) on stroke risk are discussed in the next sections.

## Data Availability

The data used to support the findings of this study are available from the corresponding author upon request.

## Supplementary Materials

Supplementary Materials Supplementary Material 1: 37 knowledge-based rules related to the 6 daily living habits (smoking, alcohol consumption, drinking tea, diet, sleep, and sport). Material 2: table of factor sets and their weights.
