# Supplementary material for: Influences of Daily Life Habits on Risk Factors of Stroke Based on Decision Tree and Correlation Matrix
Source: Comput Math Methods Med. 2020 Jun 1;2020:3217356. doi: 10.1155/2020/3217356 (PMC7285386; doi:10.1155/2020/3217356)
Supplement: Supplementary Materials — Supplementary Material 1: 37 knowledge-based rules related to the 6 daily living habits (smoking, alcohol consumption, drinking tea, diet, sleep, and sport). Material 2: table of factor sets and their weights. [file 3217356.f1.doc]

***Supplementary Materials***

**Supplementary Material 1:**

The 37 knowledge-based rules related to the 6 daily living habits (smoking, alcohol consumption, drinking tea, diet, sleep, sport):

1. SH = n + Age <= 50 + Hyte = n + Dysl = y + Sport = C1: M

*The meaning of this rule is that there is no history of stroke, younger than 50 years old, no hypertension, but dyslipidemia and less exercise. In this case, the risk of stroke is moderate.*

2. SH = n + Age <= 50 + Hyte = n + Dysl = y + Sport = C3 + Gen = F + Diab = n + FSH = n: M

3. SH = n + Age <= 50 + Hyte = n + Dysl = y + Sport = C3 + Gen = F + Diab = y: H

4. SH = n + Age <= 50 + Hyte = n + Dysl = y + Sport = C3 + Gen = M: H

5. SH = n + Age <= 50 + Hyte = n + Dysl = y + Sport = C2 + FSH = n: M

6. SH = n + Age <= 50 + Hyte = n + Dysl = y + Sport = C2 + FSH = y + Gen = F: M

7. SH = n + Age <= 50 + Hyte = n + Dysl = y + Sport = C2 + FSH = y + Gen = M: H

8. SH = n + Age <= 50 + Hyte = n + Dysl = n + Diab = n + FSH = n + TIA = n + Smok = n + Sport = C1: N

9. SH = n + Age <= 50 + Hyte = n + Dysl = n + Diab = n + FSH = n + TIA = n + Smok = n + Sport = C3 + BMIc = B3 + Gen = F + Sleep = TB: L

10. SH = n + Age <= 50 + Hyte = n + Dysl = n + Diab = n + FSH = n + TIA = n + Smok = n + Sport = C3 + BMIc = B3 + Gen = F + Sleep = TL: L

11. SH = n + Age <= 50 + Hyte = n + Dysl = n + Diab = n + FSH = n + TIA = n + Smok = n + Sport = C3 + BMIc = B3 + Gen = M + Age <= 41: L

12. SH = n + Age <= 50 + Hyte = n + Dysl = n + Diab = n + FSH = n + TIA = n + Smok = n + Sport = C3 + BMIc = B4: L

13. SH = n + Age <= 50 + Hyte = n + Dysl = n + Diab = n + FSH = n + TIA = n + Smok = n + Sport = C3 + BMIc = B2 + DT = C3 + Age <= 48: L

14. SH = n + Age <= 50 + Hyte = n + Dysl = n + Diab = n + FSH = n + TIA = n + Smok = n + Sport = C3 + BMIc = B2 + DT = C1 + Gen = F: L

15. SH = n + Age <= 50 + Hyte = n + Dysl = n + Diab = n + FSH = n + TIA = n + Smok = n + Sport = C3 + BMIc = B2 + DT = C1 + Gen = M + Alco = n: N

16. SH = n + Age <= 50 + Hyte = n + Dysl = n + Diab = n + FSH = n + TIA = n + Smok = n + Sport = C3 + BMIc = B2 + DT = C1 + Gen = M + Alco = y: L

17. SH = n + Age <= 50 + Hyte = n + Dysl = n + Diab = n + FSH = n + TIA = n + Smok = n + Sport = C3 + BMIc = B1: L

18. SH = n + Age <= 50 + Hyte = n + Dysl = n + Diab = n + FSH = n + TIA = n + Smok = n + Sport = C3 + BMIc = B5: L

19. SH = n + Age <= 50 + Hyte = n + Dysl = n + Diab = n + FSH = n + TIA = n + Smok = n + Sport = C2 + BMIc = B3: N

20. SH = n + Age <= 50 + Hyte = n + Dysl = n + Diab = n + FSH = n + TIA = n + Smok = n + Sport = C2 + BMIc = B4 + Gen = F: L

21. SH = n + Age <= 50 + Hyte = n + Dysl = n + Diab = n + FSH = n + TIA = n + Smok = n + Sport = C2 + BMIc = B4 + Gen = M + Age <= 38: L

22. SH = n + Age <= 50 + Hyte = n + Dysl = n + Diab = n + FSH = n + TIA = n + Smok = n + Sport = C2 + BMIc = B2: N

23. SH = n + Age <= 50 + Hyte = n + Dysl = n + Diab = n + FSH = n + TIA = n + Smok = n + Sport = C2 + BMIc = B1 + Age <= 48: N

24. SH = n + Age <= 50 + Hyte = n + Dysl = n + Diab = n + FSH = n + TIA = n + Smok = y + Sport = C1 + Tea = n: L

25. SH = n + Age <= 50 + Hyte = n + Dysl = n + Diab = n + FSH = n + TIA = n + Smok = y + Sport = C1 + Tea = y + BMIc = B3 + DT = C3 + Age <= 44: L

26. SH = n + Age <= 50 + Hyte = n + Dysl = n + Diab = n + FSH = n + TIA = n + Smok = y + Sport = C1 + Tea = y + BMIc = B3 + DT = C1: N

27. SH = n + Age <= 50 + Hyte = n + Dysl = n + Diab = n + FSH = n + TIA = n + Smok = y + Sport = C1 + Tea = y + BMIc = B3 + DT = C2: L

28. SH = n + Age <= 50 + Hyte = n + Dysl = n + Diab = n + FSH = n + TIA = n + Smok = y + Sport = C1 + +BMIc = B4: L

29. SH = n + Age <= 50 + Hyte = n + Dysl = n + Diab = n + FSH = n + TIA = n + Smok = y + Sport = C1 + +BMIc = B1: L

30. SH = n + Age <= 50 + Hyte = n + Dysl = n + Diab = n + FSH = n + TIA = n + Smok = y + Sport = C1 + +BMIc = B5: L

31. SH = n + Age <= 50 + Hyte = n + Dysl = n + Diab = n + FSH = n + TIA = n + Smok = y + Sport = C3: L

32. SH = n + Age > 50 + Hyte = n + Diab = n + Dysl = n + FSH = y + Smok = n + TIA = n + Sport = C1 + AF = n: M

33. SH = n + Age > 50 + Hyte = n + Diab = n + Dysl = n + FSH = y + Smok = n + TIA = n + Sport = C1 + AF = y: H

34. SH = n + Age > 50 + Hyte = n + Diab = n + Dysl = n + FSH = y + Smok = n + TIA = n + Sport = C2 + AF = n: M

35. SH = n + Age > 50 + Hyte = n + Diab = n + Dysl = n + FSH = y + Smok = n + TIA = n + Sport = C2 + AF = y: H

35. SH = n + Age > 50 + Hyte = n + Diab = n + Dysl = n + FSH = y + Smok = n + TIA = y: T

37. SH = n + Age > 50 + Hyte = n + Diab = n + Dysl = n + FSH = y + Smok = y: H

**Supplementary Material 2:**

Table of factor sets and their weights

| **Factor set** | **Number** | **Weight** |
| --- | --- | --- |
| SH,Age,Hyte,Dysl,Sport | 5 | 0.20 |
| SH,Age,Hyte,Dysl,Sport,Gen,Diab,FSH | 8 | 0.13 |
| SH,Age,Hyte,Dysl,Sport,Gen,Diab,FSH,Age | 9 | 0.11 |
| SH,Age,Hyte,Dysl,Sport,Gen,Diab | 7 | 0.14 |
| SH,Age,Hyte,Dysl,Sport,Gen | 6 | 0.17 |
| SH,Age,Hyte,Dysl,Sport,FSH | 6 | 0.17 |
| SH,Age,Hyte,Dysl,Sport,FSH,Gen | 7 | 0.14 |
| SH,Age,Hyte,Dysl,Diab,FSH,TIA,Smok,Sport | 9 | 0.11 |
| SH,Age,Hyte,Dysl,Diab,FSH,TIA,Smok,Sport,BMIc,Gen,Sleep | 12 | 0.08 |
| SH,Age,Hyte,Dysl,Diab,FSH,TIA,Smok,Sport,BMIc,Gen,Age | 12 | 0.08 |
| SH,Age,Hyte,Dysl,Diab,FSH,TIA,Smok,Sport,BMIc | 10 | 0.10 |
| SH,Age,Hyte,Dysl,Diab,FSH,TIA,Smok,Sport,BMIc,DT,Age | 12 | 0.08 |
| SH,Age,Hyte,Dysl,Diab,FSH,TIA,Smok,Sport,BMIc,DT,Gen | 12 | 0.08 |
| SH,Age,Hyte,Dysl,Diab,FSH,TIA,Smok,Sport,BMIc,DT,Gen,Alco | 13 | 0.08 |
| SH,Age,Hyte,Dysl,Diab,FSH,TIA,Smok,Sport,BMIc,DT,Gen | 12 | 0.08 |
| SH,Age,Hyte,Dysl,Diab,FSH,TIA,Smok,Sport,BMIc,DT,Gen,Age | 13 | 0.08 |
| SH,Age,Hyte,Dysl,Diab,FSH,TIA,Smok,Sport,BMIc | 10 | 0.10 |
| SH,Age,Hyte,Dysl,Diab,FSH,TIA,Smok,Sport,BMIc,Gen | 11 | 0.09 |
| SH,Age,Hyte,Dysl,Diab,FSH,TIA,Smok,Sport,BMIc,Gen,Age | 12 | 0.08 |
| SH,Age,Hyte,Dysl,Diab,FSH,TIA,Smok,Sport,BMIc | 10 | 0.10 |
| SH,Age,Hyte,Dysl,Diab,FSH,TIA,Smok,Sport,BMIc,Age | 11 | 0.09 |
| SH,Age,Hyte,Dysl,Diab,FSH,TIA,Smok,Sport,BMIc,Alco | 11 | 0.09 |
| SH,Age,Hyte,Dysl,Diab,FSH,TIA,Smok,Sport,Tea | 10 | 0.10 |
| SH,Age,Hyte,Dysl,Diab,FSH,TIA,Smok,Sport,Tea,BMIc,DT,Age | 13 | 0.08 |
| SH,Age,Hyte,Dysl,Diab,FSH,TIA,Smok,Sport,Tea,BMIc,DT | 12 | 0.08 |
| SH,Age,Hyte,Dysl,Diab,FSH,TIA,Smok,Sport,BMIc | 10 | 0.10 |
| SH,Age,Hyte,Dysl,Diab,FSH,TIA,Smok,Sport,DT | 10 | 0.10 |
| SH,Age,Hyte,Dysl,Diab,FSH,TIA,Smok,Sport,BMIc | 10 | 0.10 |
| SH,Age,Hyte,Dysl,Diab,FSH,TIA,Smok,Sport | 9 | 0.11 |
| SH,Age,Hyte,Dysl,Diab,FSH,TIA,Smok,Sport,BMIc,Gen | 11 | 0.09 |
| SH,Age,Hyte,Dysl,Diab,FSH,TIA,Smok,Sport,BMIc,Gen,DT,Tea,Alco | 14 | 0.07 |
| SH,Age,Hyte,Dysl,Diab,FSH,TIA,Smok,Sport,BMIc,Gen,DT,Tea,Alco,Age | 15 | 0.07 |
| SH,Age,Hyte,Dysl,Diab,FSH,TIA,Smok,Sport,BMIc,Gen,DT,Tea | 13 | 0.08 |
| SH,Age,Hyte,Dysl,Diab,FSH,TIA,Smok,Sport,BMIc,Gen,DT | 12 | 0.08 |
| SH,Age,Hyte,Dysl,Diab,FSH,TIA,Smok,Sport,BMIc,Alco | 11 | 0.09 |
| SH,Age,Hyte,Dysl,Diab,FSH,TIA,Smok,Sport,BMIc | 10 | 0.10 |
| SH,Age,Hyte,Dysl,Diab,FSH,TIA | 7 | 0.14 |
| SH,Age,Hyte,Dysl,Diab,Smok | 6 | 0.17 |
| SH,Age,Hyte,Dysl,Diab,Smok,Sleep | 7 | 0.14 |
| SH,Age,Hyte,Dysl,TIA | 5 | 0.20 |
| SH,Age,Hyte,Dysl,Diab,FSH,Smok | 7 | 0.14 |
| SH,Age,Hyte,Dysl,Diab,FSH,Smok,Sport | 8 | 0.13 |
| SH,Age,Hyte,Dysl,Diab,FSH,Smok,Sport,Alco | 9 | 0.11 |
| SH,Age,Hyte,Dysl,Diab,FSH,Smok,Sport,Alco,Sleep,Age | 11 | 0.09 |
| SH,Age,Hyte,Dysl,Diab,FSH,Smok,Sport,Alco,Sleep | 10 | 0.10 |
| SH,Age,Hyte,Dysl,Diab,FSH,Smok,Sport,Age | 9 | 0.11 |
| SH,Age,Hyte,Dysl,Diab,FSH | 6 | 0.17 |
| SH,Age,Hyte,Dysl,Diab | 5 | 0.20 |
| SH,Age,Hyte,Diab,Dysl,TIA | 6 | 0.17 |
| SH,Age,Hyte,Diab,Dysl,FSH,AF,TIA | 8 | 0.13 |
| SH,Age,Hyte,Diab,Dysl,FSH,AF,Tea | 8 | 0.13 |
| SH,Age,Hyte,Diab,Dysl,FSH,Smok,TIA,Sport,AF | 10 | 0.10 |
| SH,Age,Hyte,Diab,Dysl,FSH,Smok,TIA,Sport | 9 | 0.11 |
| SH,Age,Hyte,Diab,Dysl,FSH,Smok,TIA,Sport,AF | 10 | 0.10 |
| SH,Age,Hyte,Diab,Dysl,FSH,Smok,TIA | 8 | 0.13 |
| SH,Age,Hyte,Diab,Dysl,FSH,Smok | 7 | 0.14 |
| SH,Age,Hyte,Diab | 4 | 0.25 |
| SH,Age,Hyte,TIA | 4 | 0.25 |
